# Quantitative analysis of post-TAVI aortic regurgitation with cardiovascular magnetic resonance and the relationship to transthoracic echocardiography

**DOI:** 10.1186/1532-429X-15-S1-P114

**Published:** 2013-01-30

**Authors:** Akhlaque Uddin, Timothy Fairbairn, Christopher D Steadman, Bernhard A Herzog, Manish Motwani, Ananth Kidambi, Dominik Schlosshan, Daniel Blackman, Gerry P McCann, Sven Plein, John P Greenwood

**Affiliations:** 1 Academic Unit of Cardiovascular Medicine, Multidisciplinary Cardiovascular Research Centre (MCRC) & Leeds Institute of Genetics, Health and Therapeutics, Leeds, UK; 2 National Institute for Health Research(NIHR) Leicester Cardiovascular Biomedical Research Unit, University of Leicester, Leicester, UK; 3 Department of Cardiology, Leeds Teaching Hospitals NHS trust, Leeds General Infirmary, Leeds, UK

## Background

Transcatheter Aortic Valve Implantation (TAVI) is increasingly used to treat patients with severe aortic stenosis at high surgical risk. The severity of post-implantation valvular or paravalvular regurgitation has been shown to adversely affect patient outcome. The aim of the study was to assess the prevalence and severity of aortic regurgitation (AR) at 6 months post-TAVI using cardiovascular magnetic resonance (CMR).

## Methods

Twenty five severe aortic stenosis patients underwent a 1.5T CMR (Intera, Philips Healthcare) scan at baseline and 6 months after CoreValve™ TAVI. LV function was assessed using cine imaging with a steady state free precession pulse sequence. The LV outflow tract was imaged in two planes and through-plane phase contrast velocity imaging was performed perpendicular to the aortic valve and transverse to the aorta at the sinotubular junction. Post-processing was performed using QMass 7.2 and QFlow 5.2 (Medis, Netherlands). AR severity was defined using regurgitant fraction (RF) as: none to mild <8%, mild to moderate 8 to 19%, moderate to severe 20 to 29% and severe >30% [1].

Transthoracic echocardiography (iE33, Philips Healthcare) was performed at baseline and 6 months follow-up. Aortic regurgitation was graded using a comprehensive integrated approach following the recent Valve Academic Research Consortium (VARC) guidelines.

## Results

Mean age was 80.6±6.6yrs, 44% were female, Logistic EuroSCORE 19.5±14.9 LV ejection fraction significantly improved post-TAVI (52.1±11.8% vs. 55.9±9.6%, p<0.0001) and reduction in indexed end-systolic LV volume (46±18 ml/m^2^ vs. 41±17 ml/m^2^, p = 0.02). The end-diastolic volume (95±18 ml/m^2^ vs. 91±20 ml/m^2^, p = ns) and stroke volume (48±10 ml/m^2^ vs. 50±10 ml/m^2^, p = ns) did not change.

**Figure 1:**
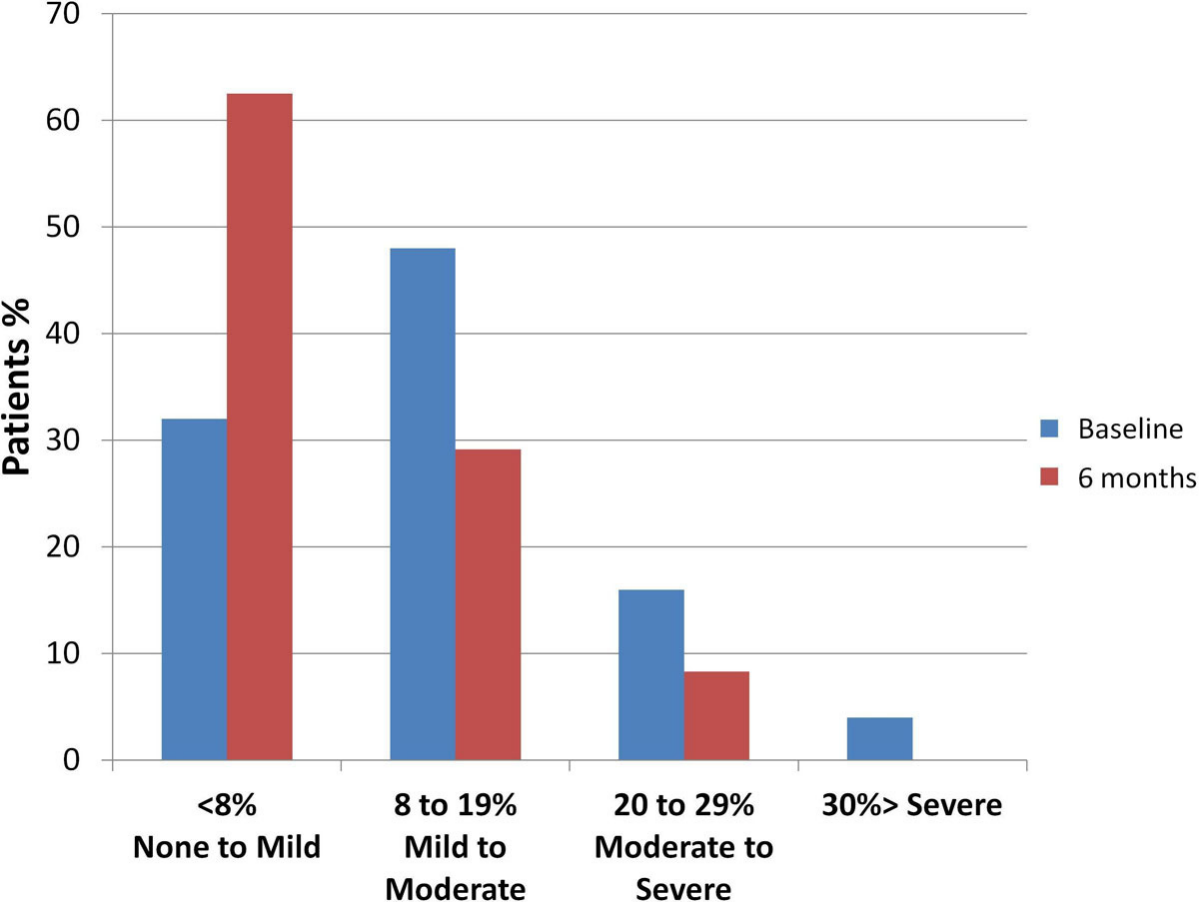
Quantification of aortic regurgitation by CMR phase contrast velocity mapping before and 6 months after TAVI implantation.

**Figure 2:**
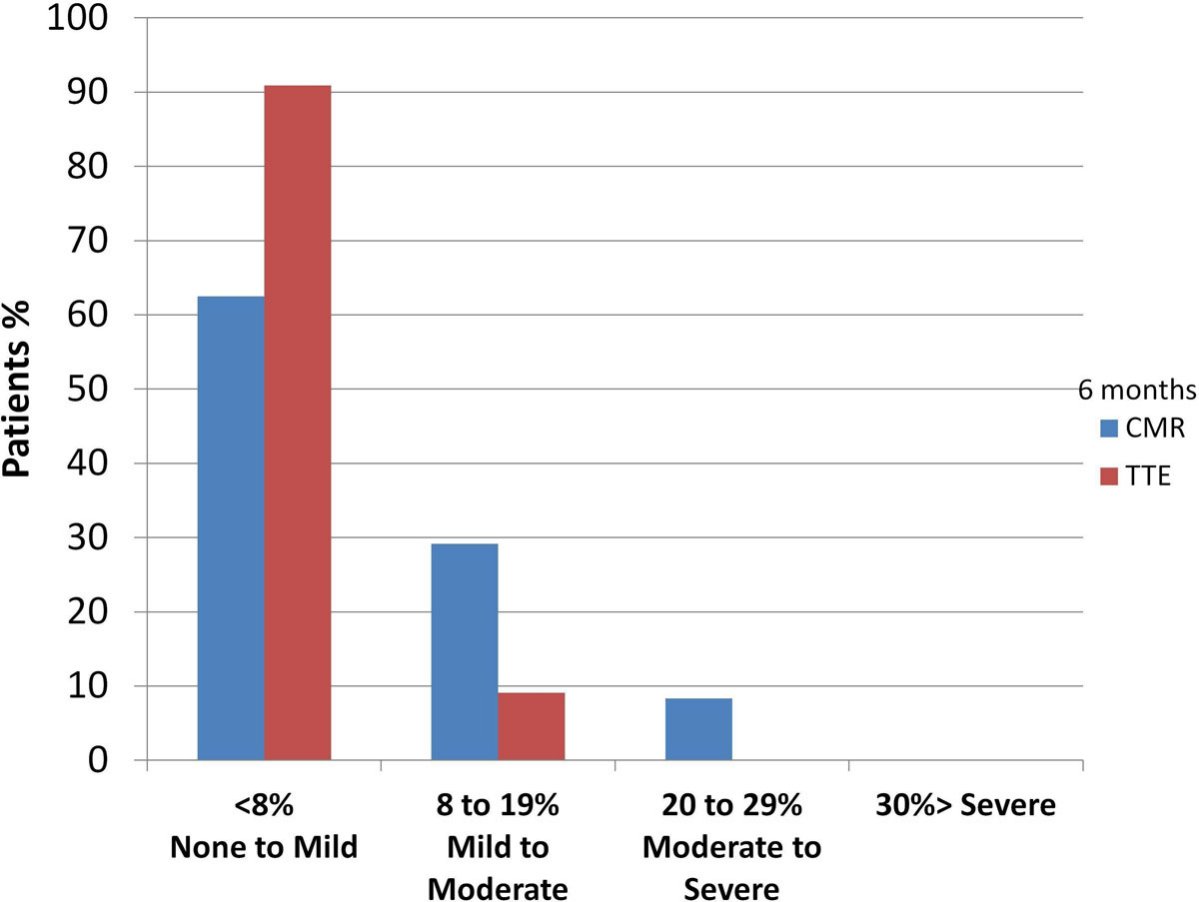
Comparison of aortic regurgitation grading by CMR and transthoracic echocardiography at 6 month follow up.

There was a significant reduction in aortic RF 6 months post-TAVI (median RF 12.4%, IQR 5.6 to 16.8% vs. 6.2% IQR 3.6 to 13.2%,p=0.034 ) (Figure 1). There was no significant difference between the transthoracic echo grading and CMR grading of aortic regurgitation. (Chi-squared = 3.74 p = 0.159) (Figure 2).

Echocardiography showed there was also a statistically significant reductions in peak forward flow velocity (4.87±0.57 ms^-1^ vs.1.98±0.35 ms^-1^ p < 0.05), peak pressure gradient (96.1±24.3 mmHg vs.17±5.7 mmHg p < 0.05) and mean pressure gradient (54.8±15.9 mmHg vs.8±3 mmHg p < 0.05) compared to baseline; the effective orifice area (EOA) was significantly larger compared to the baseline state (0.57±0.03 cm^2^ vs. 1.63±0. 3cm^2^ p < 0.05).

## Conclusions

There was an overall reduction in aortic regurgitant fraction post-TAVI even in the presence of pre-existing AR. CMR can be used in the TAVI population, pre- and post-procedure to quantify the degree of aortic regurgitation

## Funding

SP is funded by a British Heart Foundation fellowship (FS/10/62/28409).

SP and JPG receive an educational research grant from Philips Healthcare.

## References

[B1] Gabriel RS Renapurkar R Bolen MA Verhaert D Leiber M Flamm SD Griffin BP Desai MY Comparison of severity of aortic regurgitation by cardiovascular magnetic resonance versus transthoracic echocardiography Am J Cardiol 2011 108 7 1014 20 10.1016/j.amjcard.2011.05.034 21784393

